# An Escape Game on University Students’ Mental Health During the COVID-19 Pandemic: Cocreation Study

**DOI:** 10.2196/48545

**Published:** 2024-03-18

**Authors:** David Labrosse, Clara Vié, Hana Hajjam, Clément Tisseron, Dimitri Thellier, Ilaria Montagni

**Affiliations:** 1 Tricky Bordeaux France; 2 University of Bordeaux Inserm Bordeaux Population Health Research Center U1219 Bordeaux France

**Keywords:** students, game, mental health, COVID-19, cocreation, university student, promote, psychological well-being, effective tool, tool, acceptability, testing, questionnaire, learning, motivation, user centered

## Abstract

**Background:**

The COVID-19 pandemic has had a severe impact on students’ mental health. Interventions are needed to promote their psychological well-being and prevent mental illnesses in the aftermath of this unprecedented situation. Digital escape games can be an effective tool to support students’ mental health. A cocreation approach can improve the acceptability of these interventions by involving different stakeholders (eg, end users, game designers, and health professionals) to obtain audience-specific games.

**Objective:**

This study aims to describe the process of testing and optimizing the game “EscapeCovid” on students’ mental health, to serve as a model for the cocreation of future similar interventions.

**Methods:**

The PRODUCES (Problem, Objective, Design, End Users, Cocreators, Evaluation, Scalability) framework was used. Cocreation steps (test and optimization) were detailed for replicability. A total of 45 students tested a pilot version of the game, with 10 undergoing a semistructured interview. Meetings with a group of stakeholders and brainwriting were organized to optimize the game.

**Results:**

We produced a new version of the game incorporating the suggestions provided by student testers and following the stakeholders’ guidelines. Improvements were made to both the content and the form of the new version of the pilot game. The storyline, including the protagonist and the scenes, was adapted to the student population.

**Conclusions:**

Our results suggested that cocreation can contribute to the design of more widely accepted interventions aimed at promoting mental health and preventing psychological disorders. Results also suggest that an end user–centered approach can facilitate intervention tailoring. When conceiving a health-related escape game for students, we recommend using the cocreation approach to enhance players’ experience, thus positively influencing their learning process and overall well-being.

## Introduction

### University Students’ Mental Health and the COVID-19 Pandemic

University students are often likely to experience serious mental health problems during their studies because they are exposed to several stressors including academic pressure, taking on more adult-like responsibilities, or having limited financial resources [1].

The COVID-19 pandemic has exacerbated students’ mental health, as demonstrated by a skyrocketing incidence of mental health disorders during the repeated lockdowns between 2020 and 2021 [2,3]. Shifting to online courses, uncertainty about academic and professional future, and a dramatic reduction of social interactions have largely contributed to compromising the mental health of the student population [4]. Restrictive measures, in particular, were associated with high levels of depression, anxiety, and stress in students [5-7]. In 2020, nearly one-fifth of students experienced suicidal ideation as a result of COVID-19 [8]. The prevalence of these mental health problems was more than 50% higher among students than in the general population [9]. Students’ mental distress due to the COVID-19 pandemic persisted in the aftermath of the peak of the pandemic [10].

Against this background, there has been a high demand for mental health prevention programs addressed to students during and after the lockdowns [11]. Several studies have described the development and application of interventions aimed at supporting students during the health crisis. In particular, digital psychological interventions have produced positive effects on students by promoting resilience and well-being [12]. During the lockdowns, when face-to-face contact was limited, digital interventions had the advantage of reaching a larger audience with no time or space limit. Examples of interventions were video clips, online booklets, mobile applications, virtual doctor appointments, etc. University students, in particular, actively sought this type of online help and interventions, probably because they are digital natives [13].

### Gamification in Mental Health–Related Interventions

To optimize interventions, gamification is considered an essential strategy, including in the mental health field [14]. Gamification relies on the full involvement of the player and exploits several psychosocial determinants affecting the learning process (eg, self-efficacy, social interaction, and a positive learning environment) [15]. Enhancing these factors can facilitate the recall of abstract concepts, such as the concept of mental health [16]. Introducing gamified elements (eg, step-by-step sequencing, rewarding systems, and puzzle-solving activities) in a health promotion and prevention tool can influence the aforementioned psychosocial determinants and, consequently, stimulate participants’ learning loop and their cognitive capacity [17].

Over the last few years, increasing attention has been paid to the possibility of games improving well-being [18]. Games can engage players, especially young target populations, in understanding and retaining information in a more attractive and acceptable way [19]. This means that they can increase their mental health literacy and their knowledge of both the symptoms of psychological problems and the different solutions to overcome them. By providing tips and skills to face psychological difficulties, games might also contribute to positive changes in individuals’ behaviors and attitudes.

### Escape Games as a Tool to Improve Psychological Well-Being and Prevent Mental Disorders

Escape games are a type of digital intervention based on gamification where players collaborate to find clues, complete tasks, and solve puzzles with the aim of achieving a specific, time-bound goal, which is usually to escape from a room. Previous research corroborated the constructive impact of escape games in improving health-related knowledge in players [20,21] using a learning-by-doing approach [21].

Escape games can contribute to delivering health-related messages by fostering motivation for behavioral change through an enjoyable and playful approach, according to the PRIME (Plan, Response, Impulses, Motives, and Evaluation) theory of motivation [22]. Based on this theory, a decision to engage in an activity will not result in action unless it generates the desire and the impulse to do it at the relevant moment. Thus, the stimuli generated from the act of playing a game—including different tasks, lights, sounds, and colors—trigger feelings, ideas, and brain activities for positive decision-making. In other words, the game gives the input to change.

Additionally, gamification has the potential to increase motivation, engagement, and self-awareness, and even diminish symptoms of diseases such as depression and anxiety [23]. Indeed, gamification stimulates several components of good mental health. As an example, achieving goals in a game can result in a sense of satisfaction, accomplishment, and increased self-esteem, all of which improve overall well-being. Furthermore, game enjoyment is associated with positive well-being and social and emotional support [24].

### The Cocreation of Escape Games

Cocreation occurs when end users and service providers, often along with other participants, work together in the early phases of the development of an intervention cycle [25]. Cocreation is a process facilitating the acceptability of an intervention because it primarily considers the needs and preferences of end users during the intervention development. Thus, the benefits of gamification tend to increase when cocreation is used [26], and cocreating an escape game can foster its adoption [27]. Based on this approach, game producers and end users must first exchange views to achieve a shared goal [28,29]. Indeed, when developing a public health–related game, players’ experience and needs are relevant for enhancing its effectiveness in promoting health and prevention. Ideally, players work alongside designers, health professionals, and researchers, to produce the intervention. The cooperation of players and other stakeholders is therefore essential to maximize end users’ acceptability and adherence to the game. As a result, cocreation is usually recommended to produce a successful game, including in the mental health field. Accordingly, sensitive topics and taboos should be addressed using players’ words and taking into account the levels of empathy and sympathy players display during the cocreation process. Including end users with lived experience of mental health disorders promotes a deeper understanding of the game topic [30].

### The PRODUCES Framework

PRODUCES (Problem, Objective, Design, End Users, Cocreators, Evaluation, Scalability) is among the different existing frameworks facilitating the cocreation of health-related interventions [27]. It is well-known for using a systemic approach to participatory methodology. According to this framework, the *problem* is a narrowed-down behavioral issue that the researchers and the designers wish to address. The cocreation process has an *objective* (“what” and “how”) and follows a specific predefined *design*, engaging *cocreators* who represent end users (ie, a specified target population). For the latter, all characteristics must be considered, from age to socioeconomic status, to tailor the intervention coherently. Cocreated interventions can also be *evaluated* and their *scalability* can be assessed. The satisfaction of the end user as well as the effectiveness of the intervention are elements to consider for the final evaluation. A successful intervention can be scaled up to reach a wider public. Thus, the PRODUCES framework helps to guide the participatory methodology by providing specific instructions.

### Objective

The objective of this study was to describe the process of cocreation of the escape game “EscapeCovid”. The end goal of the game was to promote university students’ mental health literacy, their beliefs about mental health, management of emotions, and positive coping strategies during the COVID-19 pandemic. An applied methodology is presented here to be used as a model for cocreating an acceptable gamified mental health intervention addressed to young people. Providing this example also has the aim to illustrate one cocreation process for the benefit of other researchers and designers.

## Methods

### The First Pilot Version of “EscapeCovid”: The Escape Game “Manage Your Emotions”

“EscapeCovid” was based on an existing escape game that was used as the skeleton of the final game. “Manage Your Emotions” was created in 2021 during the pandemic by a start-up based in Bordeaux, France, specializing in producing both real-world and online escape games. Creators were game designers, programmers, and health care professionals aged under 35 years.

The game “Manage Your Emotions” is set in Tony’s room, a fictional university student living in a shared flat and experiencing the difficulties of the first lockdown. The goal of the game is to collect several tools to disclose emotion cards and combine them. The game session involves 4 players and a game guide. The role of the game guide is to coordinate the whole game, to give clues if the players are stuck, and to animate the debriefing session. The game lasts in total 2 hours: 45 minutes of play and 1 hour and 15 minutes of debriefing. During the debriefing, the game guide and the players discuss in more detail the concept of emotions (ie, how to identify and manage them). The game guide follows a predefined plot facilitating the interactions between participants.

**Figure 1:**
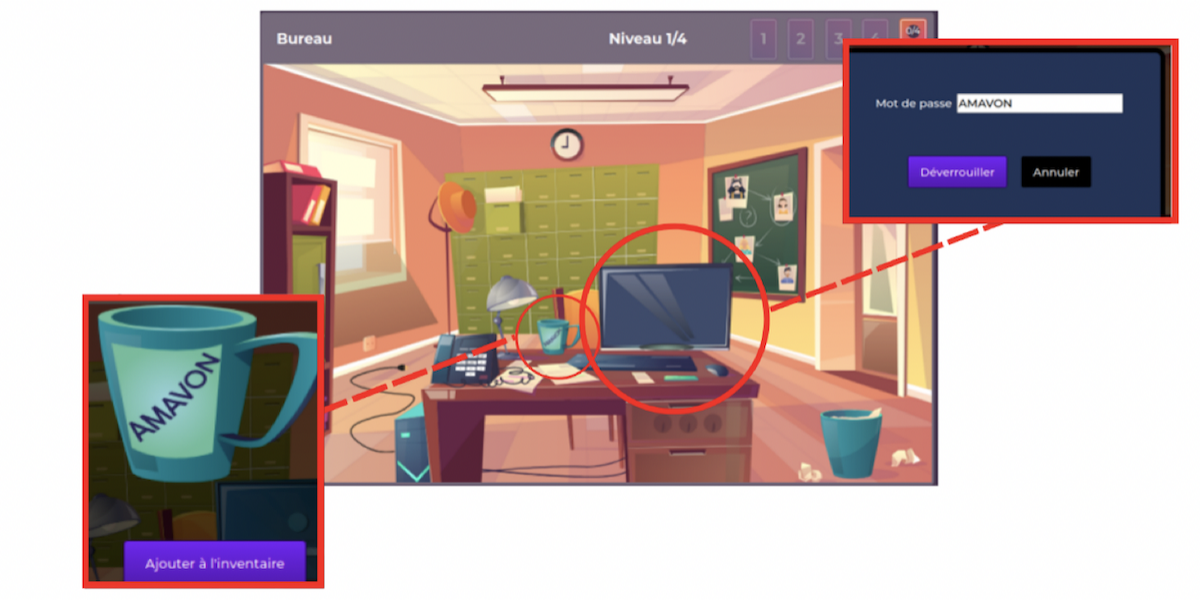
Tony's office in the escape game "Manage Your Emotions."

During the game, players follow Tony during a typical day through 3 rooms of his apartment (his home office in the bedroom, living room, and bedroom). By doing so, players discover his emotions and their consequences on his daily life. Figure 1 illustrates Tony’s home office in the bedroom with a set of clues for the players.

**Figure 2:**
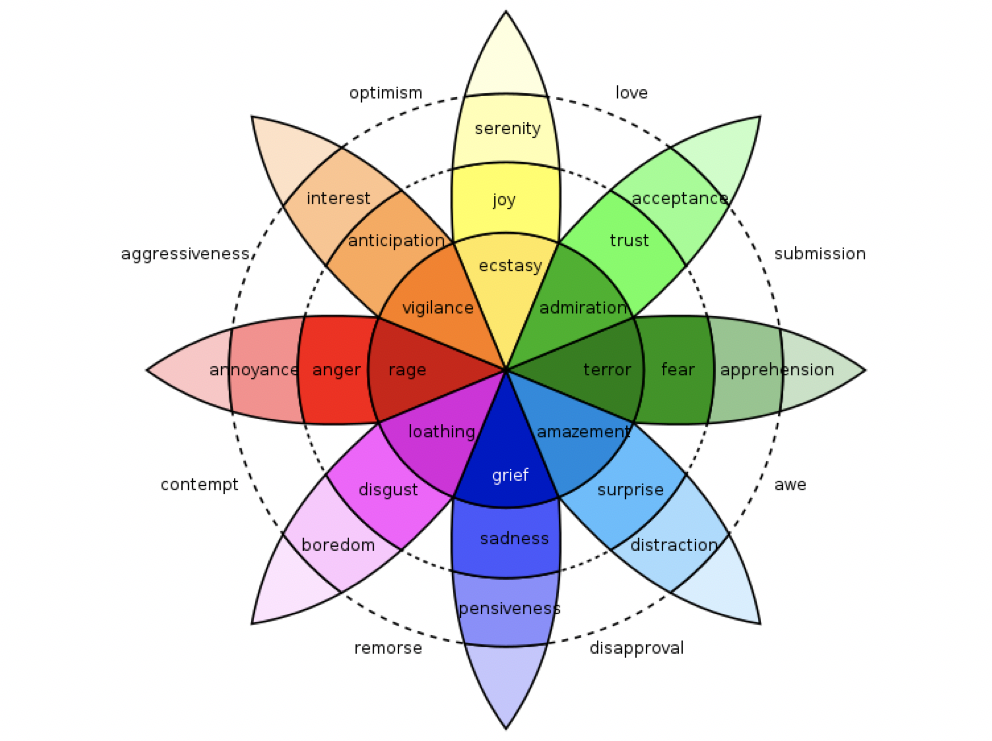
Plutchik's wheel of emotions.

Players solve puzzles by clicking on the elements on the screen to uncover emotion cards. The definition of the different emotions is based on Plutchik’s wheel of emotions [31] (Figure 2) which is the theoretical framework of the game.

Plutchik’s wheel of emotions covers 8 primary emotions: joy, trust, fear, surprise, sadness, anticipation, anger, and disgust. They can be combined into more complex secondary emotions—for example, the combination of joy and trust can result in love. In the wheel, darker colors correspond to more intense emotions. All combinations and intensities are explained in the cards. Plutchik’s theory posits that the more we know about emotions, the better we understand how various emotions are interlinked and how they can change over time. Plutchik’s wheel of emotions has been used in several studies as a scientific instrument to interpret emotions [32,33]. In this escape game, playing cards had to be associated to identify Plutchik’s emotions.

As the name suggests, the game “Manage Your Emotions” exclusively focused on emotions and therefore did not cover the full range of features of mental health (eg, mental health literacy and positive coping strategies). Furthermore, the software presented several bugs and the scenarios did not reflect the real-life conditions of a student during the pandemic.

### Cocreating “EscapeCovid”: Test and Optimization

Our cocreation process followed the PRODUCES framework [27]. The problem we chose was students’ mental health during the COVID-19 pandemic. We particularly focused on mental health literacy, beliefs about mental health, management of emotions, and positive coping strategies as the levers to act upon for increasing students’ psychological well-being. We addressed all types of mental health problems, but specifically anxiety and depression, among the most common troubles in young people [34]. These problems were exacerbated during the repeated COVID-19–related lockdowns [35].

Our objective was to develop the “EscapeCovid” game. The project was born during the third lockdown in France (from April 3 to May 3, 2021 [not included], ie, 29 days), where students were especially penalized because all educational institutions, but universities, were open. At that time, the plight of university students was prominently featured in the French media, which in turn heightened the pressure on French politicians [36].

As for the design aspect, we used a 2-step participatory methodology approach (test and optimization), as described below. Both steps involved students as players of the game before and during its improvement. Thus, through direct experimentation, cocreators were a sample of students representing all university students referred to as end users.

The evaluation was performed through questionnaires and semistructured interviews using a mixed methods approach. Students reported their opinions on the game allowing for an assessment of its qualities and defaults. In this sense, the design and the evaluation were strictly related.

In terms of scalability, our objective was to distribute the new game among additional universities catering to French-speaking students (eg, France, Africa, and Québec).

Table 1 reports the components of our study corresponding to the PRODUCES framework, including the phases and steps of the project.

**Table 1:** The PRODUCES^a^ framework applied to the “EscapeCovid” study.

PRODUCES framework	Application in "EscapeCovid"	Corresponding element/phase
Problem	To address students’ mental health during the COVID-19 pandemic	N/A^b^
Objective	To develop the “EscapeCovid” game	N/A
Design	Participatory methodology approach	Cocreation (test + optimization)
End users	Students	Optimization
Cocreators	45 health care students + 2 game guides + 1 developer + 1 project manager + 1 student intern + 1 designer+ 1 developer + 1 medical doctor + 1 researcher	Cocreation (test + optimization)
Evaluation	45 questionnaires and 10 semistructured interviews (mixed methods)	Test
Scalability	Disseminate the new game to other French students	Optimization

^a^PRODUCES: Problem, Objective, Design, End Users, Cocreators, Evaluation, Scalability.

^b^N/A: not applicable.

In practice, referring more specifically to design, cocreation was implemented following 2 steps: the test and the optimization. In the first step, the escape game “Manage Your Emotions” was tested by a sample of health care students, 2 game guides (both public health experts), and 1 developer (a computer scientist). Game guides trained with colleagues and friends to annotate their first impressions of the game flow. They commented on the difficulties encountered while animating the game, including interactions with players and technical issues. The collected information was used to improve the debriefing and to solve bugs in collaboration with the developer.

Then, the official test was launched. Health care students played the game in groups of 3 or 4, each group supervised by 1 game guide. At the end of the game session, health care students were asked to complete an online questionnaire to rate their experience and provide feedback for improvement. Semistructured interviews were conducted with some voluntary respondents to obtain more in-depth advice (for improving the game).

**Figure 3:**
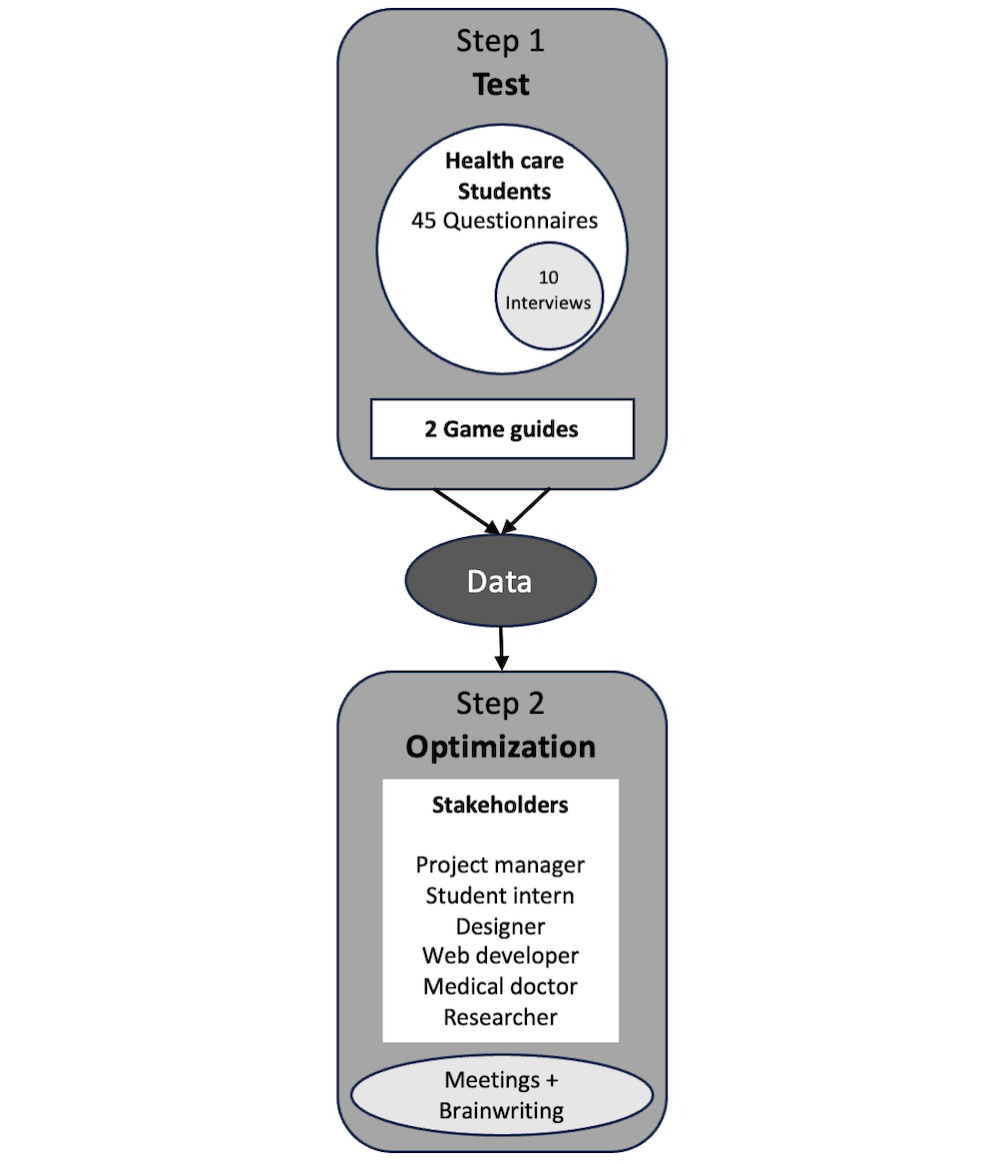
The cocreation process.

In the second step, the game was optimized using the data collected from the test with the collaboration of a group of stakeholders. The latter included 1 project manager from a public health research center, 1 student intern completing a degree in cognitive engineering, 1 game designer, the developer of the test step specialized in computer science, 1 medical doctor, and 1 psychology researcher. Meetings were organized to reshape the pilot game and brainwriting techniques were used to collect ideas and pass them on to stakeholders. The brainwriting technique involves the written generation of ideas by different individuals on separate sheets, which are then collated by the project manager in the same shared file. Ideas are categorized and synthetized in the shared document where stakeholders can discuss them with written comments and paragraphs. Then, the team meets in person to agree on a common solution. Meetings and brainwriting were done in an iterative loop [37]. Figure 3 illustrates the cocreation process we adopted.

### The Testers of “EscapeCovid”

The students testing the game were included exclusively if they were health care students registered at the University of Bordeaux (France). We accepted all specific health-related fields of study (eg, medicine, midwifery, speech therapy), genders, and ages. We opted for health care students to assess the relevance of the contents of the game given their expertise in medical and paramedical care. Furthermore, previous studies have shown that health care students are a population at risk of mental illness [38] and, as a consequence, tend to be more sensitive to this topic. Recruitment was conducted from April 27 to May 17, 2022. We used a snowball sampling approach starting with health care students doing their internship at the public health research center where the study was based. Additionally, health care–related student associations were asked to post a recruitment announcement on their social media pages. We aimed to recruit between 30 and 50 health care students, as this number would guarantee the feasibility of the study and the interactions required during the game. Thus, the recruitment was stopped when we reached a sample of 45 health care students and the recruitment strategies seemed to be no longer efficient (ie, no further responses). The final number of health care students who took part in the study was 45.

Through an email or by clicking on a link on the association’s social media posts, participants in the study were directed to a form to schedule the game session and then randomly allocated to a session including 4 players each.

All 45 health care students received a €20 (US $22) gift card. Among them, 10 also volunteered to take part in a semistructured interview and received a supplementary €20 (US $22) gift card.

In addition to health care students, testers included 2 game guides and 1 developer employed by the start-up producing the game. No inclusion or exclusion criteria were considered for these testers who were all females and aged between 25 and 30 years.

The overall aim of the project was to coproduce a game and not to measure its impact on the mental health of health care students playing the game. Students were in charge of testing the intervention, as opposed to being on the receiving end. In other words, they were not the research sample but were actively engaged in designing and implementing the research process [39]. Nonetheless, they were provided with a list of mental health care services they could refer to if needed. The medical doctor and the researcher in psychology from the stakeholders’ group were also available upon request. Finally, respondents were asked to electronically sign a consent form stating that their answers were completely anonymous without tracing. Interviewees also signed a form assuring that the recording of the interview would be deleted after 5 years until the final report and the last published paper, according to the policy of the involved research center.

### Data Collection Instruments and Analysis During the Test

A mixed methods approach was applied using both questionnaires and semistructured interviews which were administered to our sample. The satisfaction questionnaire was sent by email to students 1 day after having played the game. It was created ad hoc by IM for this study and tested with 3 public health interns at the research center where the project was conducted. The interns played the escape game and answered the questions reporting to IM if they were adapted and appropriate, and whether it was easy to answer them. Some adjustments to the original items were made after this pilot testing. The final satisfaction questionnaire included 12 items on the degree of appreciation and relevance of the intervention. On a visual analog scale from 0 (not at all) to 10 (a lot), students had to rate the game in terms of how enjoyable it was, the quality of its content, its level of difficulty, the graphics, and the clarity and relevance of the objective. Students were also asked to state to whom they would recommend the game, whether they would pay to play it, whether they had understood the importance of talking about mental health, whether the game increased their knowledge about mental health, whether the game helped them speak more freely about mental health, and whether they felt the game destigmatized mental health. Participants were also asked to rate the game from 1 (very bad) to 5 (very good) stars. We included these specific questions because they provided concrete hints for improving the game. The start-up appraised the features with the lowest scores as the most important to consider when reshaping the game. For instance, they chose to work first and foremost on the graphics if players rated them low (ie, <5 points). Some questions helped understand if the game could have its own business model, with players advising and paying for it. Finally, the questions were aimed at assessing the impact the game had on students’ mental health literacy, ranging from destigmatization to readiness to seek help [40]. The items of the satisfaction questionnaire are available in Multimedia Appendix 1. Sociodemographic characteristics were also collected, including students’ gender, age, and year of study. Variables were described as counts and percentages. The questionnaire allowed us to obtain a large number of answers in a short time from a young population that is often difficult to reach [41].

Semistructured interviews were based on a grid composed of 3 macro themes and related 13 subthemes. The first macro theme, called “General Description of the Participant” included the following 3 subthemes: students’ profile (sociodemographic characteristics), any previous experience with escape games, and the reason for participating in this study. The second macro theme was a “Brief Account of Participants’ Experience” during the game session of “Manage Your Emotions,” focusing on 5 subthemes: whether students enjoyed it, their satisfaction with the design and scenarios, the feasibility of the game, the learning outcomes, and any advice to improve the game. The last macro theme, “The Impact of the Game,” included questions on the effectiveness of the game in teaching 5 topics (each corresponding to a specific subtheme): mental health, stigmatization, understanding and managing emotions, the importance of help-seeking, and techniques for mental health promotion. All subthemes were applied deductively, meaning they had been determined before the interviews.

Then, individual students’ speeches were generalized to obtain an overall assessment of the game. Interviews were recorded, fully transcribed, and analyzed through qualitative coding. The framework method was used to cross-check results among individuals and within individuals to report common and consistent concepts [42]. This approach allowed us to list the guidelines for the optimization step.

### Ethics Considerations

As the goal of the project was to collect satisfaction data with no repercussions on participants’ health, no ethical approval was needed, in line with the French law for health-related research (Délibération n° 2018-155 du 3 mai 2018).

## Results

### Sociodemographic Characteristics

The quantitative sample of 45 students was purposely limited for a small-scale test. Among them, 34 were female students, 10 male, and 1 nonbinary. Their average age was 22 years (range 18-27 years). The years of study ranged from first-year students to PhD candidates, with the majority attending their fourth year (n=16). The qualitative sample (n=10) was composed of 7 female students and 3 male students. In the qualitative study, one-half of the sample declared having experienced a mental health problem and having seen a mental health specialist.

### Students’ Gaming Experience

The sample of 45 students who answered the questionnaire and the sample of 10 students interviewed reported enjoying the game session.

We discuss between us, why and how it is this emotion and not another [...] it was really good.

B, female PhD candidate, Public Health, first year

Yeah, I really liked the associations of emotions [...] frankly, we spoke with people we didn't know, so frankly it went well, it was cool.

D, male students, Pharmacy, fourth year

The majority of the sample (30/45, 67%) gave a high score (between 8 and 10) when asked whether they enjoyed the game. For 34/45 (76%) it was interesting (scores from 8 to 10). Twenty-one students considered that the game was easy. The most frequent overall score given to the game was 4 out of 5 stars (25/45, 56%).

In line with this finding, 21/45 (47%) respondents gave a positive score between 8 and 10 regarding the appeal of the graphics. Concerning the visual staging of the game, 1 student declared that the storyline was not coherent:

Tony’s apartment is too big for being a student flat.

MA, female student, Speech Therapy, second year

Tony’s character was also discussed, with some students questioning his relatability:

When Tony was talking, I didn't really get into the thing, in the end I found it very tricky, too tricky, a bit like a fake student.

L, female student, Speech Therapy, third year

Regarding the overall content of the game, 27/45 (60%) participants found the objective of the game clear (scores from 8 to 10) and 30/45 (67%) considered the content of the game suitable for students (scores from 8 to 10).

Half of the sample (24/45, 53%) would recommend the game to their close friends and family, especially their friends attending university (43/45, 96%). However, the vast majority of students (36/45, 80%) would not pay to play it.

### The Knowledge Acquired During the Online Game Session

For 27/45 (60%) of the respondents, the game made them understand the importance of talking about mental health and 38/45 (84%) thought that the game was likely to increase their knowledge about mental health. However, interviewed students reported that knowledge about mental health was addressed in an unsuitable way.

For students, the game enabled users to better understand and identify different emotions, but the general concept of mental health was missing:

It was really more about identifying emotions, and self-reflection.

L, female student, Speech Therapy, fourth year

There would be a wealth of important information to address on mental health.

A, female student, fourth year of international health

### The Development of the “EscapeCovid” Game

Quantitative and qualitative data from the test (step 1) informed the optimization of the game “Manage Your Emotions” (step 2) to produce the new game “EscapeCovid”. Data were collated and analyzed by the stakeholders working on the development of the game. All results were considered to reshape the new escape game accordingly. The results of the mixed methods analyses were shared among stakeholders. This group of experts met 3 times to summarize the most important suggestions provided by the testers. Each meeting lasted from 3 to 4 hours. Then, 1 shared document was prepared and the stakeholders were asked to provide solutions for each suggested change. This was the beginning of the brainwriting process, where stakeholders updated the document once per week and met regularly every other week. Ideas were incorporated into a new working document, which was the basis for a new round of discussions (ie, five 2-hour meetings). Once consensus had been reached, the web developer revised the game following the guidelines written by the stakeholders on an online document and Figma (Figma, Inc.), a collaborative web application for interface design. Both the contents and the designs were discussed and modified.

Table 2 reports the modifications made from the first version of the game to the final one (also see Textboxes 1 and 2 for the topics addressed and educational content of versions 1 and 2).

**Table 2:** Comparison of features from the 2 versions of the game.

Features	Version 1: “Manage Your Emotions”	Version 2: “EscapeCovid”
Objectives	To teach players to name, identify, and manage their emotions.	To increase students’ knowledge of mental health by familiarizing them with a range of emotions and symptoms of depression and anxiety.
Game flow	45-minute game session (3 rooms: home office in the bedroom, living room, and bedroom) + 45-minute debrief.	Alternating game/debrief sessions in each room (home office in the bedroom, living room, and bedroom) and evaluation questions.
Topics addressed and educational content	See Textbox 1.	See Textbox 2.
Character(s)	Tony, a confined student.	Thomas, a confined student. Thomas’ roommate, “Hana.” A researcher who appears on the screen to give instructions and clues if the players need them.
Team competition	Accumulation of points assigned according to the speed with which the player solves puzzles.	Accumulation of points based on different criteria: speed in solving a puzzle, number of clicks used, time spent in each room and in the entire game, and correct answers to evaluation questions. Teams can also lose points if they choose to access clues to solve puzzles or if they answer assessment questions incorrectly.
Storyline	Tony is a student living confined in his shared flat during the first lockdown. Game users follow him and the emotions he felt throughout lockdown. Players must solve puzzles to access emotion cards.	The same story plot as version 1. The presence of a new character changes the transition from 1 room to the other. Players must solve puzzles to access emotion cards.
Game setting	The graphics of the game are similar to an apartment of a young worker and not a student. The vocabulary used by Tony is not adapted to the target audience.	A student flat share; instead of having a home office in the bedroom and a separate 2-bedroom flat, the home office in the bedroom is on one side of Thomas’ room. Thomas’ voice and vocabulary have also been adapted to meet the expectations/requests of the target audience.

**Topics addressed and educational content of version 1: “Manage Your Emotions.”**

1. Game
  - This involved knowledge, identification, and management of emotions.
2. Debriefing Session
  - This involved tips and resources to identify and manage emotions.
  - In addition, the emotions felt during the examination period were discussed.

**Topics addressed and educational content of version 2: “EscapeCovid.”**

1. Game (examining symptoms of depression through emotions):
  - Stress and anxiety: other emotions, fear.
  - Anhedonia: sadness.
  - Self-devaluation: disgust.
  - Other topics related to mental health that were addressed, including the following:
    - Names of some mental health illnesses.
    - Stigmatization of mental health and mental illness.
    - Resources
2. Debriefing session
  - Room 1: Home office in the bedroom within the bedroom
    - Stress and anxiety: definitions and differences. Link with depression.
  - Room 2: Living room
    - Relationship between mental health and anhedonia. Link with depression.
  - Room 3: Bedroom
    - Relationship between mental health and self-devaluation. Association with depression.
3. Debriefing/end of the game
  - Emotions not previously addressed during the game.
  - Experienced symptoms and depression.
  - Stress on the importance of having good mental health.
  - Resources available in the case of a mental health problem.

**Figure 4:**
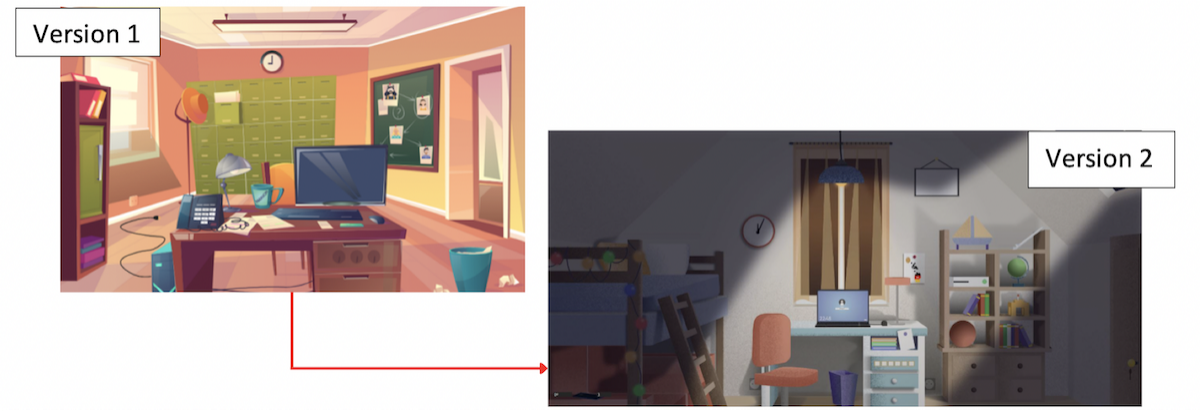
Evolution of the graphics of the first room of "Manage Your Emotions" versus "EscapeCovid".

In particular, substantial modifications concerned the introduction of mental health–related information in the game. Students had confirmed that the first version of the game was exclusively oriented toward emotions and their management. There were no specific elements of mental health described as either mental diseases or psychological well-being. In “EscapeCovid,” the terms “depression” and “anxiety” were used by the main character. The symptoms and consequences of these mental health diseases were presented in the story. Depression and anxiety were selected because they are the most frequent psychological issues among young adults [43]. Players were supposed to learn more about mental health problems (mental health literacy), to destigmatize them (positive beliefs about mental health), and be able to tackle them (positive coping strategies). Puzzles and enigmas were used to teach these concepts with debriefing sessions to reinforce the learning process. Given their expertise in the field of health care, interviewees (health care students) helped with the writing of the plot, from the enigmas to the summary sheets. An example of a cocreated scenario within the story is available in Multimedia Appendix 2. Thanks to a cocreation approach, the game content could be revised by all public health stakeholders who were experts in public health and psychology. The graphics were also modified as shown in Figure 4.

### Playing the “EscapeCovid” Game

The escape game takes place in Thomas’ apartment which he shares with another student, Hana, during the first COVID-19 lockdown. Thomas is a university student and is taking his classes remotely. Throughout the game, we follow him during a typical day in lockdown. There are 3 rooms in Thomas’ apartment—a home office in the bedroom, a living room, and a bedroom. To move from 1 room to another, players must solve all the puzzles by clicking on the objects spread out in Thomas’ room. When players click on an object, a riddle appears and must be solved to move on to the following riddle. Players can only move to another room if they have solved all of the enigmas by uncovering clues or cracking the codes hidden in the sofa, among books, on the floor, etc. There is a limited number of clicks per participant.

At the end of each room scenario, a set of cards is shown, with each card containing a mental health–related message linked to the puzzles. For instance, in the living room, Hana is sleeping on the sofa in the dark and the books around her have titles containing the words “depression,” “pain,” etc. By solving clues and clicking, players can switch the light on and tidy up the room to make her recover strength. The cards synthesize the messages transmitted through the puzzles in the room. In this case, they explain the symptoms of depression and give tips for coping with distress.

“EscapeCovid” can be played in groups of 4-6 players who help each other and discuss using their computer cameras and headphones. This encourages team spirit and mutual aid, which can be the reflection of real life in the case of mental suffering. All along the game, the group of players is guided by a game guide who explains the rules and answers any questions. The same guide concludes the game session with a final debrief where all participants share their experiences. This final stage is essential for understanding and retaining the mental health–related takeaway messages.

## Discussion

### Principal Findings

We described the process we used to cocreate a digital game promoting students’ mental health during the COVID-19 pandemic. We followed a 2-step procedure. First, we collected quantitative and qualitative data from a manageable sample of students testing a preexisting game. Second, a group of stakeholders used these data to refine and optimize the game to obtain the final user-centered version.

The cocreation approach was very informative for developing the “EscapeCovid” game. In particular, during the test, students felt free to express their opinions openly and give feedback. They mostly appreciated the fact that they could support the development of an intervention addressed directly to them and their peers. Students were also motivated to cocreate the game because it was in line with their values. Students’ contribution to the design process nurtured new ideas following a collective creative approach [44] from the testing phase to the final optimization phase. Stakeholders’ work was facilitated by students’ guidelines while being creative and innovative.

### The Rationale and the Usefulness of “EscapeCovid”

During the COVID-19 crisis, several mental health diseases emerged in the young, and digital games were among the most accepted solutions to overcome psychological difficulties [45]. With this rationale, we conceived "EscapeCovid". This game was designed to alleviate anxiety and depression by encouraging interaction with peers and fostering empathy. Participants in this study also confirmed the usefulness and appreciation of digital games during and after the COVID-19 crisis. Previous studies have shown that playing games is helpful in dealing with trauma and improves well-being [46]. This has also been observed in the context of the pandemic [47]. For this, "EscapeCovid" combines the pleasure and the entertainment of games, with a positive psychological effect. This might be due to teamwork, engagement, learning of coping strategies, and creativity, which are all at the root of our game. Indeed, the objective of "EscapeCovid" was to trigger the need to speak out about mental health after having experienced the psychological difficulties of COVID-19. "EscapeCovid" pioneered the discussion of mental health, making it a common topic, and provided advice on how to improve one’s mental health, especially in the aftermath of the crisis.

### Guidelines for Successful Escape Games on Young’s People’s Mental Health

First, we confirmed that students enjoy playing escape games, which are linked to mental health. This was also found in other studies where health-related serious games were proven to facilitate experiential learning through an entertaining approach [21,48]. Thus, resorting to this type of intervention could be a good strategy to convey messages aimed at improving players’ mental health. Engaging in playing games has been reported to promote the potential to enhance life satisfaction and improve individuals’ mental well-being [18].

We observed that the plot was essential in capturing players’ attention. During the game, testers were attracted by the messages and the scenario, feeling interested in following Thomas’ story. They considered this aspect as crucial to transmit educational content, helping to convey new health-related topics, as shown in a previous study [21]. A meta-analysis on the gamification of learning confirmed that the use of personified narrative components is particularly effective in promoting behavioral learning [49].

Playing in groups was also a strategy to make connections and combat isolation, particularly experienced during the COVID-19 lockdown. The notion of interrelationship and mutual aid is a component to consider when developing games, even if they have a digital format.

According to testers, the “EscapeCovid” game had to be user-friendly, fun, and pedagogical. It had to present supplementary contents on mental health, with more specific details on mental health disorders and advice for preventing or treating them. We recommend that future game creators use precise and detailed content, providing accurate and uncensored mental health information and avoiding stigmatizing psychological disorders.

### The Challenges of Cocreating Mental Health–Related Games With and For Young People

The involvement of end users entails a large proportion of subjectivity. This is especially true when handling topics such as mental health where feelings and emotions are at stake. End users give their opinions without any specific framework [50]. To mitigate this issue, the sample answering the questionnaire should be large enough to be representative of the target population. However, for the sake of feasibility in terms of time and financial efforts, it is not always possible to question more than 50-100 people. Qualitative interviews are meant to provide further information corroborating the quantitative data, but they still imply subjectivity. Per se, interviews cannot be representative [51]. The limited number of stakeholders has its share of arbitrariness. Nonetheless, regardless of their number, cocreators are the bridge between the whole target population and the stakeholders [27].

Cocreation is time-consuming. The 2-step development demands at least twice as long as the standard time to produce a game. Data collection and analysis add work to the producers who need to incorporate the results into their creative process. Discussions among stakeholders and brainwriting also slow down the production process. This is a limit of cocreation which cannot be overcome while being the best solution for producing an intervention that is well-tailored to the needs of the end user. Qualitative interviews in particular require time and effort, but they are a crucial tool for an in-depth analysis.

Technological issues should also be considered. Players’ expectations might not be easily met because of software limitations. This could result in frustration from both parties and decrease adherence to the game. Start-ups and game industries should therefore keep up with new technologies and continuously update their services.

### The Advantages of Cocreation

Cocreation has the advantage of considering the viewpoint of the end user, which might not be the case in classical processes of game development using a top–down approach. Collecting students’ opinions before the development of the game allowed us to obtain several inputs and ideas that a limited number of web developers and project managers could not provide. The filter of expert health care professionals was also essential during the process. New knowledge was produced through sharing among parties.

The consultation with students having experienced mental health disorders allowed us to address the escape game’s topic through a different lens. By considering their opinion, the game could be made more realistic and engaging. The disclosure of emotions and opinions can be facilitated through anonymous questionnaires and qualitative interviews, with students knowing that they are contributing to an intervention beneficial to them and their peers. The feeling of being useful to the community is another added value in the cocreation process [52].

Two different teams—one from an academic environment and the other from a start-up—collaborating to develop the game was also an asset. Indeed, researchers’ scientific point of view informed the business goal of the start-up with the common will of creating an evidence-based marketable product.

Finally, cocreation provided useful information for the improvement of both the content and the format of the game. The latter was more contextually specific, adapted to a young population, namely, students, and bridging the gap between the preconceived ideas held by the start-up team and the real-world implementation of the game.

### Recommendations for Researchers and Designers

The “EscapeCovid” game is an example of a digital game on mental health, which could be cocreated with young users. The guidelines we present might be applied to other similar interventions.

A 2-step approach is recommended with (1) an initial collection and analysis of combined quantitative and qualitative data, followed by (2) the integration of these data into the reflective and creative work of a group of experts and stakeholders. This approach, similar to a market survey, allows us to obtain clearer game instructions and broader insights, resulting in a more targeted and audience-specific final product.

We suggest basing the coconstruction process on an already existing pilot version because it facilitates the development of the final game. Although the game can be completely re-created, preliminary mock-ups will allow to save time and money. In our study, students were not required to design the game from scratch, and working on the first version of the game was an advantage for providing relevant, concrete, and realistic comments based on an existing version.

Stakeholders are also advised to take into account testers’ opinions seriously and implement them accordingly. Testers represent the end users and their preferences must be carefully considered to obtain a fully satisfactory end product. For this reason, it is essential to collect as much information as possible during the testing stage.

We suggest to try out the game again once it has been modified. An iterative loop of test-optimization-test will increase the quality of the game. However, it must be pointed out that this process is expensive and time-consuming, despite being extremely informative. It is therefore recommended to end the loop once the comments are saturated, which effectively means limiting the number of additional changes suggested by users and resulting work for developers. This approach can serve as a blueprint for future work on creating gamified interventions on health-related topics addressed to students. Successfully cocreated games can have a wide outreach and improved scalability.

### Study Limitations

Testers were mostly student interns at the research center where the study was conducted. This might have biased the results because participating students were already made aware of the project and willing to contribute to its progress as members of the same research laboratory. The test was performed by health care students, meaning that the modifications of the game might be relevant to them and not to students of other subjects. This particular student population also faces specific forms of stress not experienced by their peers. However, we considered their opinion to be of paramount importance in terms of the contents of the game, which benefited from their skills and experience with stress. The game was more realistic and other students could relate to Thomas’ story imagined by young people their age.

Another limitation is that the students participating in the study were rewarded with a gift card, which could have significantly influenced the answers due to desirability bias. This phenomenon was even more likely in the case of interviewees who had received 2 gift cards. The gender balance among the participants was skewed in favor of female students, which may have influenced the results of the test.

Finally, we were not able to retest the game after its modification. Because of money and time constraints, we only produced a new version of the game without further refinement.

### Conclusions

Our results suggest that cocreation contributes to improving the suitability of a health promotion and disease prevention intervention and that an end user–centered approach can facilitate intervention tailoring. When conceiving a health-related escape game, we recommend using a 2-step approach, including an initial collection of quantitative and qualitative data from end users testing the game (test), followed by the integration of these data into the development of the game by a restricted number of experts (optimization). This approach can serve as a model for future work on creating gamified interventions on health-related topics addressed to students.

## Appendix

Multimedia Appendix 1 Satisfaction questionnaire.

Multimedia Appendix 2 Example of a cocreated scenario in "EscapeCovid".

## Abbreviations

PRIME: Plan, Response, Impulses, Motives, and Evaluation
PRODUCES: Problem, Objective, Design, End Users, Cocreators, Evaluation, Scalability
