# Supplementary material for: An Escape Game on University Students’ Mental Health During the COVID-19 Pandemic: Cocreation Study
Source: JMIR Serious Games. 2024 Mar 18;12:e48545. doi: 10.2196/48545 (PMC10985612; doi:10.2196/48545)
Supplement: Multimedia Appendix 1 [file games_v12i1e48545_app1.docx]

| **YOUR OPINION ABOUT THE GAME**  This part of the questionnaire allows us to collect your opinion about playing an online session of our Escape Game  ***Your experience with the game***  **1) Was the game fun?**  0 (Not fun at all) … 10 (Very fun)  **2) Globally, is the content of the game interesting?**  0 (Not interesting at all) … 10 (Very interesting)  **3) Was the game difficult?**  0 (Not difficult at all) … 10 (Very difficult)  **4) How do you rate the graphics of the game?**  0 (Not appealing at all) … 10 (Very appealing)  **5) Is the objective of the game clear?**  0 (Not clear at all) … 10 (very clear)  **Your advice on the capacity of the game to be engaging**  **6) Is the content of the game appropriate for students?**  0 (Not appropriate at all) … 10 (Very appropriate)  **7) Would you advise those around you to play this game?**  Absolutely not  No  Maybe  Yes  Absolutely  **8) To whom would you advise the game?**  My students  My friends who are not students  My family  My parents  **9) Would you pay for a session of the game?**  Yes  Maybe  No  ***The impact of the game on your behavior***  **10) This game has allowed me to recognize the importance of mental health.**  Absolutely not  No  Maybe  Yes  Absolutely  **11) This game can increase my knowledge about mental health.**  Absolutely not  No  Maybe  Yes  Absolutely  **12) This game has allowed me to talk more freely about mental health.**  Absolutely not  No  Maybe  Yes  Absolutely  **13) This game can destigmatize mental health.**  Absolutely not  No  Maybe  Yes  Absolutely  **14) This game can promote my intention to ask for help concerning my mental health.**  Absolutely not  No  Maybe  Yes  Absolutely  **7.15. This game can improve players’ mental health.**  Absolutely not  No  Maybe  Yes  Absolutely  ***Your general advice***  **16) Which is your final note of the game?**  From 1 to 5  **17) Since having played the game, do you think you will consult one or more of the following healthcare professionals?**  Psychologist  Psychiatry  Psychiatry nurse  Psychotherapist  Other  None |
| --- |
