# Supplementary material for: An Escape Game on University Students’ Mental Health During the COVID-19 Pandemic: Cocreation Study
Source: JMIR Serious Games. 2024 Mar 18;12:e48545. doi: 10.2196/48545 (PMC10985612; doi:10.2196/48545)
Supplement: Multimedia Appendix 2 [file games_v12i1e48545_app2.docx]

In the first room scenario, the emotion cards unlocked are “anxiety”, “fear”, “despair” and “guilt”. To each of these emotion cards, the player must attach a secondary emotion. For example, the secondary emotion to relate to “anxiety” is “anticipation”. At the end, the game guide debriefs with the players who must discover the main emotion of the room scenario. “Fear” is the emotion of the first room:

Anxiety = Fear + Anticipation

Fear = Fear + Surprise

Despair = Fear + Sadness

Guilt = Fear + Joy


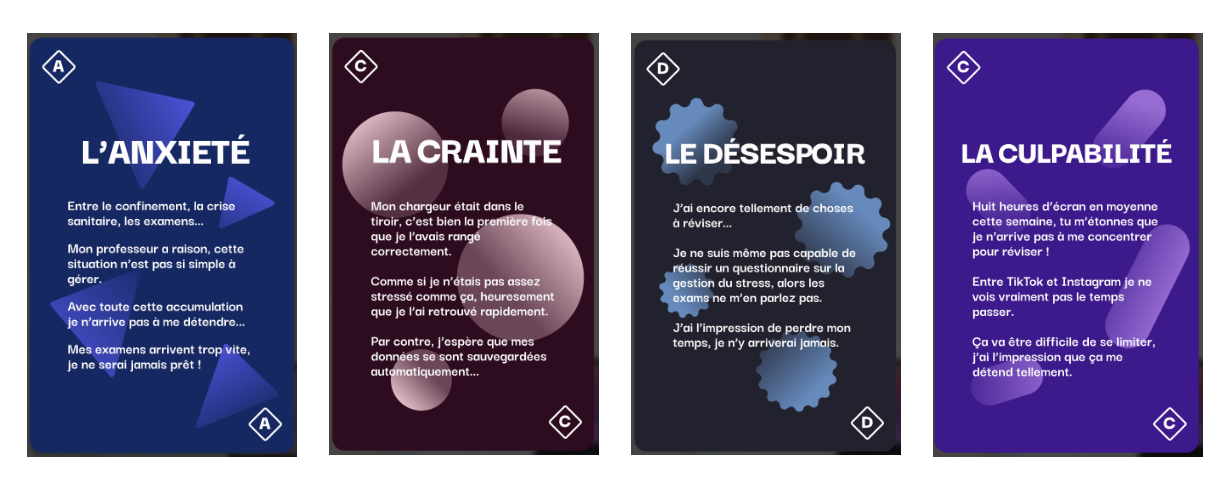


DESPAIR

GUILT

FEAR

ANXIETY

Before the players go into the second room, the game guide illustrates how to distinguish the notions of stress and anxiety. Thus, (s)he explains that stress is a natural reaction of the body to a negative or unwanted situation. It can be positive or negative, it occurs at a specific time and can be quickly relieved. Anxiety is characterized by the appearance of excessive and difficult to control worries, often accompanied by physical symptoms. It is rather present in the long term, without a solution to palliate it quickly, and can appear with no particular reason. Tony's situation in this first piece of EscapeCovid shows the accumulation of future threats such as exams, the spread of the epidemic and the continuity of restrictions, which nourish this fear. Then, players are reminded that if they are concerned or know a person suffering from anxiety, they must not hesitate to consult or advise to consult a health professional.
